# Continuously Adjustable, Molecular-Sieving “Gate” on 5A Zeolite for Distinguishing Small Organic Molecules by Size

**DOI:** 10.1038/srep13981

**Published:** 2015-09-11

**Authors:** Zhuonan Song, Yi Huang, Weiwei L. Xu, Lei Wang, Yu Bao, Shiguang Li, Miao Yu

**Affiliations:** 1 Department of Chemical Engineering, University of South Carolina, Columbia, SC 29208; 2 SmartState Center of Catalysis for Renewable Fuels, University of South Carolina, Columbia, SC 29208; 3 College of Applied Science and Technology, Rochester Institute of Technology, Rochester, NY 14623; 4 Gas Technology Institute, 1700S. Mount Prospect Road, Des Plaines, IL 60018.

## Abstract

Zeolites/molecular sieves with uniform, molecular-sized pores are important for many adsorption-based separation processes. Pore size gaps, however, exist in the current zeolite family. This leads to a great challenge of separating molecules with size differences at ~0.01 nm level. Here, we report a novel concept, pore misalignment, to form a continuously adjustable, molecular-sieving “gate” at the 5A zeolite pore entrance without sacrificing the internal capacity. Misalignment of the micropores of the alumina coating with the 5A zeolite pores was related with and facilely adjusted by the coating thickness. For the first time, organic molecules with sub-0.01 nm size differences were effectively distinguished via appropriate misalignment. This novel concept may have great potential to fill the pore size gaps of the zeolite family and realize size-selective adsorption separation.

Zeolites/molecular sieves are one of the most promising adsorbents that may help realize true molecular-sieving separation, because of their uniform, molecular-sized pores (0.3~1.3 nm) and high chemical, thermal, and mechanical stabilities^1^. Pore size gaps, however, exist in the current zeolite family, which leads to the difficulty in separating molecules with small size/shape differences, especially at the 0.01 nm level.

Pore size of zeolites/molecular sieves can be adjusted by several techniques, including ion exchange^2^, framework control^3^,^4^,^5^,^6^,^7^, and zeolite external surface modification^8^,^9^,^10^. Ion exchanges have been used as an effective way of adjusting the pore sizes of LTA (Linde Type A) zeolites^2^. The framework of some zeolites, such as zeolite rho, may deform substantially upon adsorption of some molecules^3^. A molecular sieve, ETS-4, has been shown to contract gradually through dehydration at elevated temperatures so that its effective pore size can be adjusted at approximately 0.01 nm step^4^. Recently, a novel method, called ADOR (assembly-disassembly-organization-reassembly), was applied to chemically selectively remove germanium from germanosilicate zeolite UTL in a top-down strategy to prepare a series of IPC zeolites with continuously tuneable surface area and micropore volume^5^,^6^,^7^. Pore opening size of mordenite zeolite was reduced at 0.1 nm level by chemical vapor deposition (CVD) of silica coatings on the external surface of zeolites^8^. The CVD modified ZSM-5 zeolite showed increased shape selectivity of xylene isomers, and HZSM-5 zeolite showed enhanced para-selectivity in the methylation of toluene^9^,^10^. But, the pore opening reduction mechanism for CVD modified zeolite was not clear^8^,^9^,^10^. Despite a large selection pool of zeolites/molecular sieves and available techniques to adjust their pore sizes, not all desired pore sizes can be obtained for target separations. This is especially the case for separating molecules that are very close in size. In addition, pore modification and structure changes were always realized by sacrificing adsorption capacity or internal cavity^4^,^11^,^12^,^13^. Here, we report, for the first time, a bottom-up approach for precise pore mouth size adjustment for 5A zeolite from 0.5 to 0.46 nm without sacrificing internal cavity by pore misalignment; organic molecules with size differences as small as 0.01 nm were effectively distinguished by appropriate misalignment.

We used molecular layer deposition (MLD) to form a conformal hybrid aluminum alkoxide (alucone) coating on the 5A zeolite surface (Supplementary Materials). The hybrid alucone coating was subsequently calcined in air to remove the organic compound to generate a porous alumina coating^14^. MLD provides exquisite control of the coating thickness at the sub-nanometer level and thus achieves conformal coating on substrates even with high-aspect-ratio features^15^,^16^,^17^,^18^,^19^. Figure 1a shows a transmission electron microscopy (TEM) image of 5A zeolite with 60 cycles of MLD; after calcination an approximately 20 nm thick coating was deposited on the 5A zeolite surface, corresponding to a nominal porous alumina deposition rate of 0.33 nm/cycle. The weight percentage of 60 cycles of MLD coating on 5A zeolite is estimated to be < 2% by applying the coating density^19^ and thickness, 5A zeolite solid density^20^, and external surface area of 5A zeolite crystals, estimated from the average particle size and shape (Figure S1 in Supplementary Materials). X-ray photoelectron (XP) spectra (Fig. 1b) shows after 120 cycles of MLD, silicon (2p binding energy at 102.3 eV) in 5A zeolite can hardly be seen due to the shorter excited electron mean free path than MLD coating thickness; the MLD coatings are composed of alumina (Table S1 and Figure S2 in Supplementary Materials). X-ray diffraction (XRD) confirmed LTA zeolite structure before and after MLD, and MLD coatings did not change zeolite structure (Figure S3 in Supplementary Materials). Brunauer–Emmett–Teller (BET) measurement and N_2_ sorption analysis show that 5A zeolites with and without MLD coatings had almost identical surface area (343.5 ± 8.3 m^2^/g) (Fig. 1c), and identical micropore volume (0.20 cm^3^/g) (Fig. 1c); argon sorption analysis further confirms there is no change in micropore volume after MLD coating deposition (Figure S4e). This suggests coatings were only on the external surface of 5A zeolite and the internal cavity of the zeolite was maintained. We also measured vapor adsorption isotherm of the MLD precursor, trimethyl aluminum (TMA), and found negligible adsorbed amounts (Figure S6 in Supplementary Materials). Therefore, MLD coatings are expected to be only on the external surface of 5A zeolite, instead of inside the zeolite pores. To further confirm the ultrathin MLD coating is on the 5A zeolite surface and has negligible effect on the internal cavity of 5A zeolite, we also measured CH_4_ adsorption isotherms on 5A zeolite and 5A zeolite with different cycles of MLD coatings (Fig. 1d); almost identical CH_4_ adsorbed amounts were found, indicating ultrathin MLD coating did not enter zeolite internal pores. These results demonstrate that ultrathin, porous MLD coatings were deposited only on the external surface of 5A zeolite.

We measured vapor adsorption isotherms of organic molecules with different sizes/shapes (critical diameter: ethanol, 0.450 nm; 1-propanol, 0.456 nm; 1-butanol, 0.463 nm; acetone, 0.479 nm; and 2-propanol, 0.490 nm (Figure S7 in Supplementary Materials)) to explore the effective pore sizes (Figure S8 in Supplementary Materials). Figure 2a shows the sorption capacity of different molecules on 5A zeolite and 5A zeolite with different cycles of MLD coatings, corresponding to different coating thicknesses. 5A zeolite adsorbs all these molecules because its pore size is larger than them. Also, 5A zeolite shows low ideal adsorption selectivity for these molecules due to the small size differences of these molecules and the similar adsorption strength, with the highest for ethanol over acetone (~4). We found these organic molecules, from the largest molecule (2-propanol) to the second smallest molecule (1-propanol), were excluded from the zeolite pores one by one with the increase of MLD cycles, indicating effective pore size decreased gradually. Figure 2b summarizes the pore size change with the coating thickness, and a clear gradual decrease trend of the pore size can be seen. This demonstrates the effective pore size can be precisely controlled at a step change of approximately 0.01 nm by controlling the coating thickness or MLD cycles. In addition, sorption capacity of the smallest molecule, ethanol, decreased only approximately 15% with the increase of MLD cycles up to 60, while rejecting larger molecules. Two most probable mechanisms may result in the observed effective pore size reduction: 1) the decreasing micropore size in the MLD coating with the increase of MLD cycles; 2) the reducing interface pores between the MLD coating and the 5A zeolite pores due to the pore misalignment, as depicted in Fig. 2c, which slightly and gradually reduces the zeolite pore entrance size or forms a molecular “gate” at the entrance. We propose pore misalignment as the pore reduction mechanism, whereas the first possibility is much less likely based on more evidences discussed in the next paragraphs. In addition, due to the amorphous feature of the MLD coating, we expect the extent of pore misalignment is not exactly the same above all the zeolite pores, and thus a pore entrance size distribution is likely.

We conducted a series of experiments to investigate the pore reduction mechanism, as discussed below. Figure 3a shows that after pre-adsorbing 2-propanol, the CH_4_ adsorbed amounts on 5A zeolite with 30 cycles of MLD coating (5A-Zeolite-30, abbreviation will be used in the following description) did not change and were essentially the same as those on bare 5A zeolite. However, 5A zeolite, after pre-adsorbing 2-propanol, had negligible CH_4_ uptake. Apparently, without the microporous coating 5A zeolite pores have been occupied by 2-propanol, while with 30 cycles of MLD all the zeolite pores are available for CH_4_ adsorption. This is consistent with the result in Fig. 2a, which shows 5A-Zeolite-30 successfully excluded 2-propanol. Although pre-adsorbed 2-propanol had negligible effects on equilibrium CH_4_ uptake, it drastically influenced CH_4_ uptake kinetics. Figure 3b shows that CH_4_ uptake rate is almost the same on 5A zeolite and 5A-Zeolite-30. This makes sense because the “gate” size (>0.47 nm) of 5A zeolite with ultrathin MLD coating is too large to affect the CH_4_ (kinetic diameter: 0.38nm^21^) uptake rate. After pre-adsorbing 2-propanol on 5A-Zeolite-30 for 90 minute, CH_4_ uptake rate decreased approximately 50 times, as suggested by the diffusivity difference, calculated from the short time update results^22^. Since 2-propanol can’t enter zeolite pores of 5A-Zeolite-30, the drastically slowed CH_4_ uptake rate must be due to the blocking of MLD coating pores by pre-adsorbed 2-propanol. Therefore, we conclude MLD coating pores must be larger than 2-propanol, but the “gate” size is smaller than 2-propanol so that it can’t enter 5A zeolite pores. When a much larger molecule, 2, 2-dimethylbutane (DMB) (kinetic diameter: 0.63 nm^23^), was used to pre-adsorb on 5A-Zeolite-30, the CH_4_ uptake rate was hardly influenced. Apparently, DMB can’t be adsorbed in the MLD coating pores and thus had negligible influence on CH_4_ uptake. In these uptake experiments, the equilibrium CH_4_ adsorbed amounts (M_∞_) for 5A zeolite, 5A-Zeolite-30, and 5A-Zeolite-30 after pre-adsorbing 2-propanol and DMB were very close to each other (0.70, 0.68, 0.66 and 0.69 mmol/g, respectively). Therefore, comparison of CH_4_ uptake processes is fair. We also measured ethanol adsorption kinetics on 5A zeolite and 5A zeolite with different cycles (30 and 60) of MLD coatings (Figure S9). Much slower ethanol uptake rate after MLD coating was observed, but different thickness of MLD coatings had negligible effect on ethanol uptake rate. This again suggests the major transport resistance is not in the MLD coating layer.

To further rule out the possibility that the narrowest pore or transport resistance is at the external surface of the MLD coatings or in the MLD coatings, we crushed samples with 60 cycles of MLD coating (5A-Zeolite-60C) in an attempt to damage the MLD coating. TEM image (Figure S10a in Supplementary Materials) showed that after crushing the MLD coating was partially damaged and showed irregular surface morphology. XP spectra (Figure S10b in Supplementary Materials) showed drastically increased amount of exposed silicon, compared with that without crushing. This again suggests the damage of MLD coating and thus is consistent with the TEM image. However, vapor adsorption isotherms of 2-proponal on 5A-Zeolite-60C was essentially the same as that before crushing (Figure S11 in Supplementary Materials). This indicates crushing damaged the MLD coating but did not change the interface between the MLD coating and 5A zeolite. Apparently, the narrowest pores are neither on the external surface of MLD coatings nor in the MLD coatings, but at the interface of the MLD coating and zeolite pores. These adsorption kinetics and equilibrium results strongly support that the narrowest size of MLD coated 5A zeolite must be at the interface between the MLD coating and the 5A zeolite pores and pore misalignment is the actual mechanism. We speculate the extent of misalignment is related with the thermal stress generated at the interface during calcination. Analytical modelling study^24^ has shown that interfacial shear stress due to thermomechnical loading increases with the increase of the adhesive/coating thickness, and thus larger shift/displacement is expected with thicker coatings (also see analysis in Figure S12 in Supplementary Materials).

The designable 5A zeolite with desired molecular-sieving “gate” offers a new opportunity for separating small organic molecules based on size differences as small as 0.01 nm. Figure 4a shows adsorption isotherms of ethanol and 1-butanol on 5A-Zeolite-60, and an ideal selectivity as high as ~196 has been obtained, in strong contrast with ~4 for 5A zeolite, showing its potential for extracting ethanol from 1-butanol, for example, in catalytic conversion of ethanol into 1-butanol^25^,^26^,^27^,^28^,^29^. In addition, 5A-Zeolite-30 may be used for 1-butanol/acetone separation (Fig. 4b) in the second important large-scale industrial fermentation, acetone butanol ethanol (ABE) fermentation^30^. 5A-Zeolite-20 may be used for acetone/2-propanol separation (Fig. 4c) in the hydrogenation of acetone to 2-propanol^31^. These examples suggest 5A zeolite can be rationally designed via appropriate pore misalignment by MLD to obtain desired molecular “gate” sizes for size-selective adsorption separation. We also measured 50/50 ethanol/butanol liquid mixture adsorption on 5A zeolite and 5A-Zeolite-60 and found that the adsorbed phase contains ~10% butanol in 5A zeolite but <0.5% in 5A-Zeolite-60, indicating great potential of MLD coated 5A for molecular-sieving separation of liquid mixtures.

In summary, we demonstrated a completely new concept, pore misalignment, to form a size-screening “gate” on the 5A zeolite surface. The size of the “gate” can be adjusted by changing microporous alumina coating thickness, whereas the internal cavity of zeolites will be maintained. This novel concept has great potential to be utilized to fill pore size gaps of the zeolite family and to design zeolite-based molecular-sieving sorbents for selective separation of molecules with very small size differences and may potentially be used for size-selective catalysis using zeolites/molecular sieves.

## Methods

The alucone MLD coatings were prepared by using trimethyl aluminum and ethylene glycol as precursors. Adsorption isotherms were measured by a volumetric method, using a home-built adsorption system. The BET surface areas were measured by a Micromeritcs ASAP 2020 unit. A Zeiss Ultra Plus FE-SEM was used to determine 5A zeolite particle size and morphology. X-ray powder diffraction (XRD) was carried out using a Rigaku MiniFlex II diffractometer with Cu Kα radiation. XP spectra analysis was performed using a monochromatic Al Ka x-ray source. TEM images of samples were recorded using a Hitachi H8000 TEM instrument. Further details on the experimental methods can be found in the Supplementary Information.

## Additional Information

**How to cite this article**: Song, Z. *et al.* Continuously Adjustable, Molecular-Sieving “Gate” on 5A Zeolite for Distinguishing Small Organic Molecules by Size. *Sci. Rep.* **5**, 13981; doi: 10.1038/srep13981 (2015).

## Supplementary Material

Supplementary Information

## Figures and Tables

**Figure 1:**
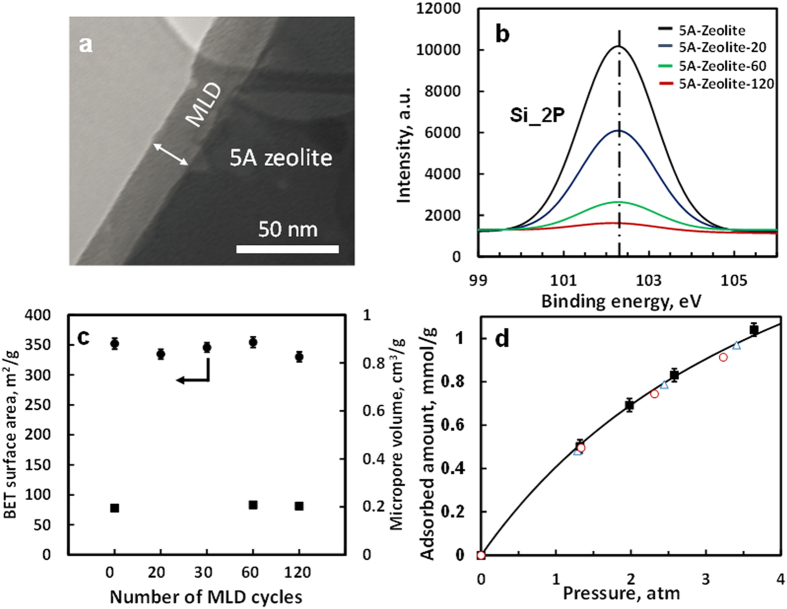
Characterization of 5A zeolite and 5A zeolite with molecular layer deposition (MLD) coatings. (**a**) Transmission electron microscopy (TEM) image of 5A-Zeolite-60. (**b**) X-ray photoelectron spectra (XPS) of Si 2P of 5A zeolite and 5A zeolite with different cycles of MLD coating on 5A zeolite. (**c**) BET surface area of 5A zeolite and 5A zeolite with different cycles of MLD coatings (•), and micropore volume of 5A zeolite and 5A zeolite with different cycles of MLD coatings (**■**). Error bar is given automatically by Micromeritcs ASAP 2020 unit. (**d**) CH_4_ adsorption isotherms at 20 °C on 5A zeolite (**■**), 5A-Zeolite-30 ( ○), and 5A-Zeolite-60 (∆). Solid black line is a fit of adsorption points of CH_4_ on 5A zeolite by the Langmuir model. All MLD coatings have been calcined in air following the procedure described in the supplementary information.

**Figure 2:**
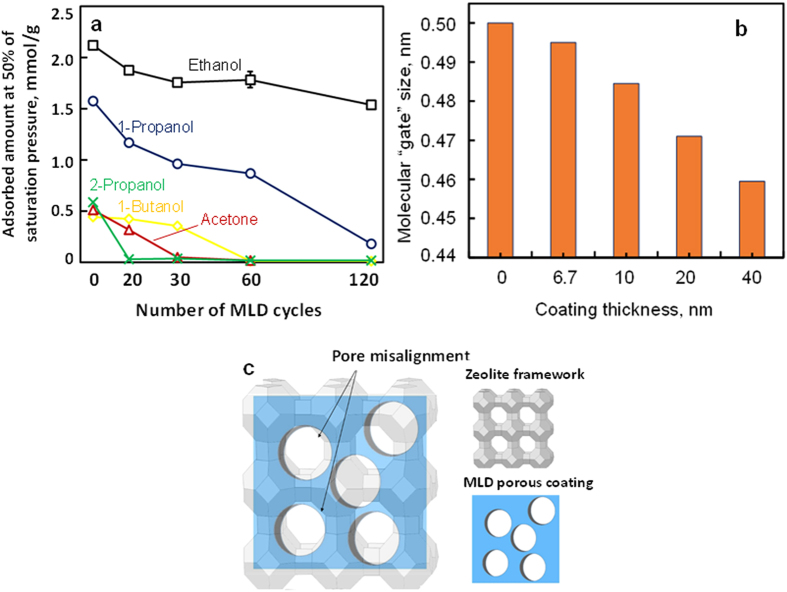
Exclusion of organic molecules with different sizes by 5A zeolite and 5A zeolite with MLD coatings. (**a**) Adsorption capacity of molecules on 5A zeolite and 5A zeolite with different cycles of MLD coating: ethanol (□), 1-propanol ( ○), 1-butanol (
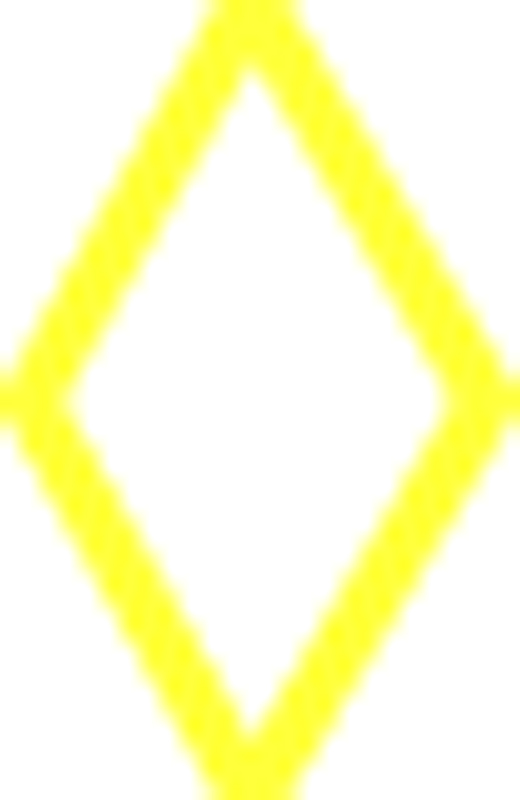
), acetone (
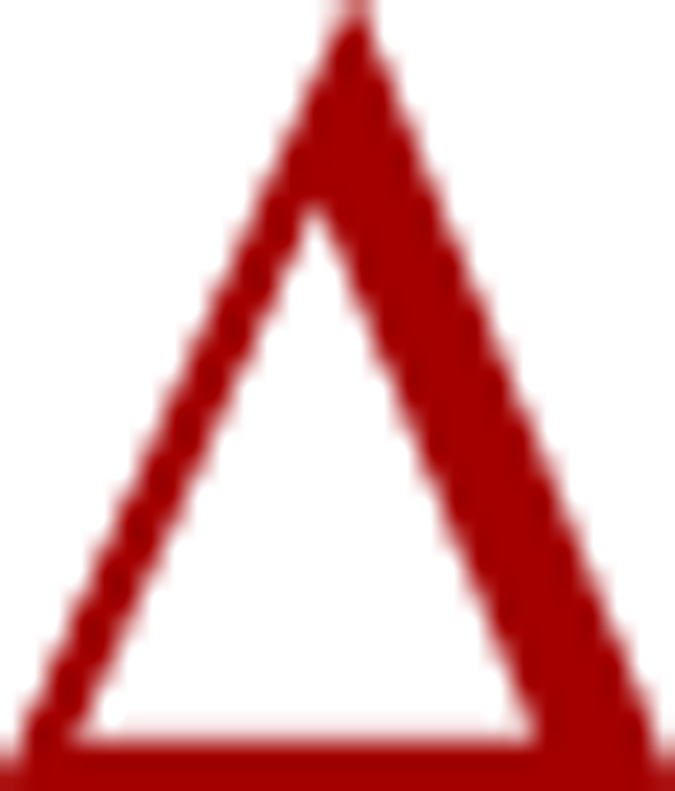
), and 2-propanol (
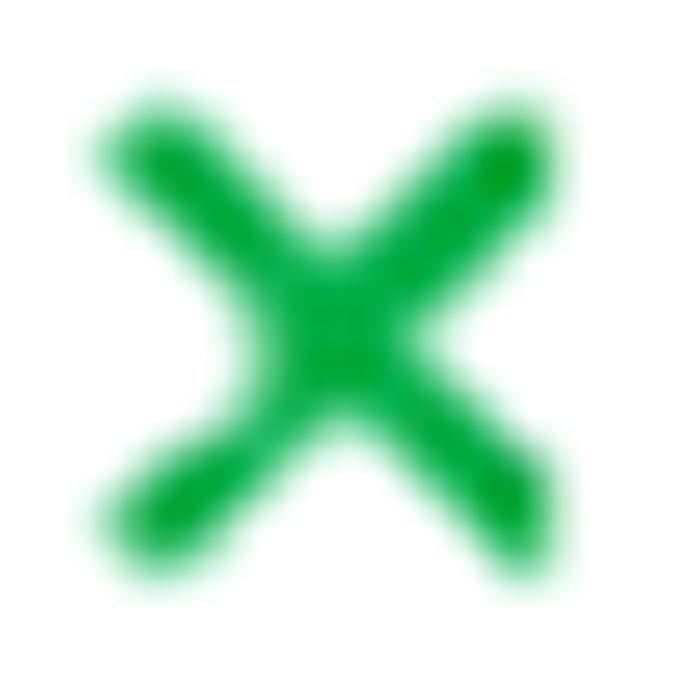
); equilibrium pressure is at 50% of the saturation pressure of each component. Error bar shows standard deviation of triplicate measurements. (**b**) Molecular “gate” sizes with different thickness of microporous alumina coatings; the “gate” size is defined as the average of the smallest excluded molecule and the largest molecule that can be adsorbed; an excluded molecule is defined as a molecule whose adsorbed amount is less than 10% of that in 5A. (**c**) Schematic representation of the proposed pore size reduction mechanism: misalignment of the micropores of the MLD coating with 5A zeolite crystal pores.

**Figure 3:**
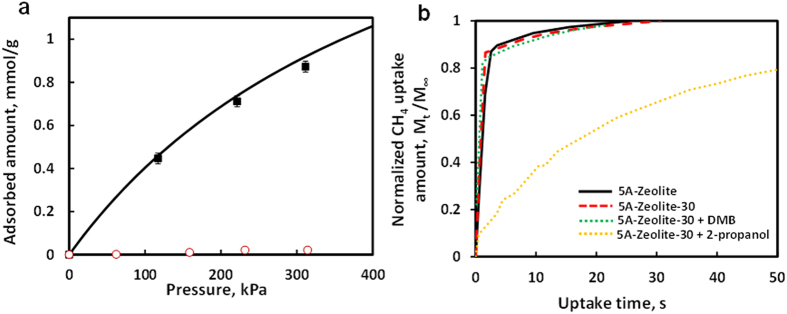
Adsorption isotherms and kinetics of CH_4_ on 5A zeolite and 5A-Zeolite-30 at 20 °C. (**a**) Adsorption isotherms of CH_4_ at 20 °C on 5A zeolite (solid black line), 5A zeolite with pre-adsorbed 2-propanol (
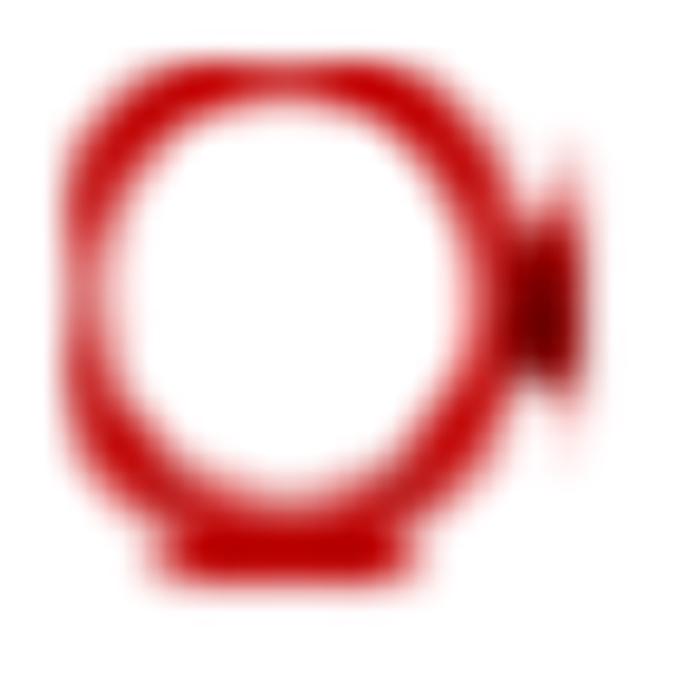
), and 5A-Zeolite-30 with pre-adsorbed 2-propanol (■). Error bar shows standard deviation of triplicate measurements. (**b**) CH_4_ adsorption kinetics on 5A zeolite (solid black line), 5A-Zeolite-30 (dash red line), 5A-Zeolite-30 pre-adsorbed with 2,2-dimethyl butane (dot green line), and 5A-Zeolite-30 pre-adsorbed with 2-propanol (dot yellow line); M_t_ is adsorbed amount of CH_4_ at time t, and M_∞_ is adsorbed amount at equilibrium.

**Figure 4:**
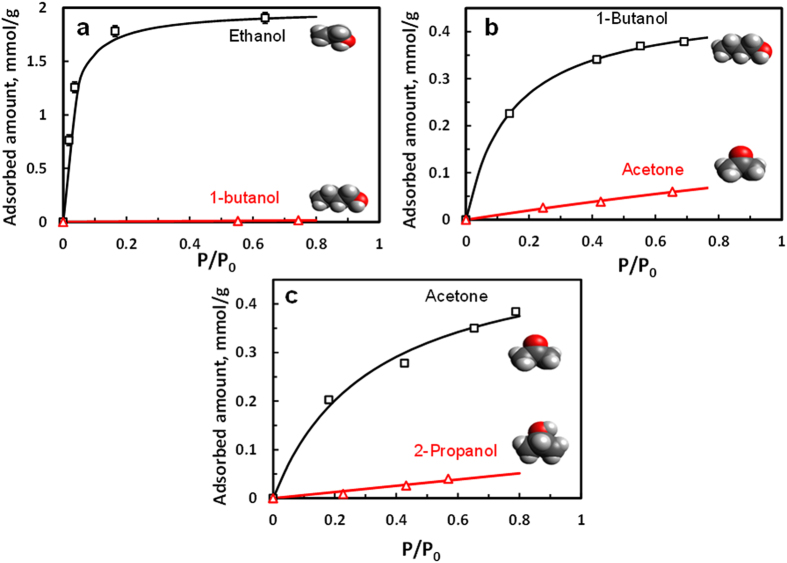
Adsorption isotherms of organic molecules at 20 ^°^C on 5A zeolite with different cycles of MLD coatings. (**a**) ethanol and 1-butanol on 5A-Zeolite-60; Error bar shows standard deviation of triplicate measurements. (**b**) 1-butanol and acetone on 5A-Zeolite-30; (**c**) acetone and 2-propanol on 5A-Zeolite-20. P is the vapor pressure, and P_0_ is the saturation pressure. P_0(Ethanol) _= 50 Torr, P_0(1-Butanol) _= 7 Torr, P_0(Acetone) _= 201 Torr, P_0(2-Propanol) _= 36 Torr.
