# Supplementary material for: Continuously Adjustable, Molecular-Sieving “Gate” on 5A Zeolite for Distinguishing Small Organic Molecules by Size
Source: Sci Rep. 2015 Sep 11;5:13981. doi: 10.1038/srep13981 (PMC4650327; doi:10.1038/srep13981)
Supplement: Supplementary Information [file srep13981-s1.pdf]

Supplementary Materials for

Continuously Adjustable, Molecular-Sieving "Gate" on 5A Zeolite for Distinguishing Small Organic Molecules by Size

Zhuonan Song, Yi Huang, Weiwei L. Xu, Lei Wang, Yu Bao, Shiguang Li, and Miao Yu

Corresponding author: Yumiao@cec.sc.edu
Co-corresponding author: Shiguang.Li@gastechnology.org

This PDF file includes:

Materials and Methods
Supplementary Text
FiguresS1 to S12
References

Materials and Methods

Molecular layer deposition (MLD) on 5A zeolite

We used 5A zeolite pellets from W.R.Grace & Co.-Conn. Zeolite pellets were firstly outgassed at 200°C for 4 h. The alucone MLD coatings were prepared by using trimethyl aluminum (TMA) ($Al(CH_3)_3$; 97%, Sigma Aldrich) and ethylene glycol ($HO(CH_2)_2OH$; 99%, Alfa Aesar) as precursors. Each MLD cycle started with 240 s vacuum. Ethylene glycol was then introduced into the reactor until a pressure of 50 mTorr and settled for 120 s, and then 240 s vacuum was followed to evacuate extra unreacted ethylene glycol. Ultrahigh purity N_2 (Airgas) was used as the purge gas at 20 sccm for 30 second. Then 240 s vacuum was applied to evacuate N_2. After that, TMA was fed into the reactor until a pressure of 300 mTorr and then settled for 120 s, followed by 240 s vacuum to evacuate extra unreacted TMA. Ultrahigh purity N_2 (Airgas) was again used as the purge gas at 20 sccm for 30 second. Then 240 s vacuum was applied to evacuate N_2. This whole process finishes one MLD cycle. MLD reactions were conducted at 100 °C. Then the coated samples were heated in air from room temperature to 250°C at a rate of 1°C/min, kept at 250°C for 4 h, and then cooled to room temperature at the same rate.

Adsorption equilibrium and kinetics measurements

Adsorption isotherms of ethanol (99.5%, Sigma-Aldrich), 1-propanol (99.5%, Alfa Aesar), 1-butanol (99%, Sigma-Aldrich), acetone (99.5%,VWR) and 2-propanol (99%, Sigma-Aldrich) on 5A zeolite and MLD coated 5A zeolite were measured by a volumetric method using a home-built adsorption system. Sorbent (~0.20 g) was firstly outgassed at 200℃ for 2 h. Helium was then used to calibrate the volume of adsorption cell with sorbent at 20℃. Commonly, helium is assumed not to adsorb and used as an inert molecule for volume calibration.[1] After removing residue helium in the adsorption system, interested vapors were introduced at 20℃ to measure the adsorption isotherms. All the vapor adsorption isotherms are shown in Figure S8. 5A zeolite adsorbs all the molecules because its pore size is larger than them. But, for MLD coated 5A zeolite, the effective pore sizes gradually decreases with the increase of MLD cycles, and thus rejected molecules one by one starting from the largest 2-propanol. Specifically, 5A-Zeolite-20, 5A-Zeolite-30 and 5A-Zeolite-60 almost completely rejected 2-propanol, acetone, and1-butanol, respectively, while 5A-Zeolite-180 almost excluded 1-propanol. Adsorption isotherm of TMA on 5A zeolite at 20℃ is shown in Figure S6. Adsorption kinetics was measured by monitoring vapor pressure change with time. For the pre-adsorption process, we followed the same procedure for organic vapor adsorption, and then introduced CH_4 for its adsorption isotherm measurements and adsorption kinetics measurements with the presence of organic vapor.

Adsorption kinetics profiles (M_t/M_∞ vs. time) of ethanol on 5A zeolite and MLD coated 5A zeolite were shown in Figure S9. M_t is the mass adsorbed at time t, and M_∞ is the mass adsorbed at infinite time. In these experiments, the values of M_∞ for 5A zeolite, 30, and 60 cycles of MLD coated 5A zeolite were18.8, 19.2 and 18.1 mg/g, respectively. Bare 5A zeolite showed much faster uptake of ethanol than that of MLD coated 5A zeolite. However, ethanol uptake rate did not change much as the MLD cycle number increased. The ethanol diffusivity in 5A zeolite and MLD coated 5A zeolite were estimated by fitting the uptake data with a simplified solution given by Kaerger and Ruthven for short times[2]:

$$\frac{M_t}{M_\infty} = \frac{6}{\sqrt{\pi}}\sqrt{\frac{Dt}{r^2}}$$

Where D is the Fickian diffusivity, and r is the edge length of the cubic crystal. A Fickian diffusivity of 1.354×10^{-14} m^2/s was obtained for ethanol in 5A zeolite. In contrast, the diffusivity

of ethanol in 30 cycles of MLD coated 5A zeolite(1.710×10^{-17} m^2/s) and 60 cycles of MLD coated 5A zeolite (1.613×10^{-17} m^2/s) were similar and about three orders of magnitude lower than that in bare 5A zeolite. This suggests MLD coating does not dominate ethanol update process. These results demonstrate that the ethanol diffusion kinetics was drastically affected by the interface between the MLD coating and 5A zeolite but not the MLD coating thickness.

Liquid mixture adsorption measurements

5A zeolite pellets (~2.0 g) and 60 cycles of MLD coated 5A zeolite pellets (~2.0 g) were firstly outgassed at 200°C for 4 h. Then the adsorbents were sealed immediately in two 20 ml vials to avoid water uptake. After cooling down to room temperature, a 50/50 (mole ratio) ethanol/butanol liquid mixture was added into a microliter vial then placed in the 20 ml vials with 5A zeolite and 60 cycles of MLD coated 5A zeolite. Parafilms were used to wrap tightly outside the 20 ml vials to prevent vapor evaporation. Liquid mixture samples were analyzed after adsorption at room temperature for 30 h by a 5890 SERIES II Gas Chromatography (GC) equipped with a 30 meter long, 0.25mm internal diameter Stabilwax column.

Molecular dimension simulation

All the models of molecules (ethanol, 1-propanol, 1-butanol, acetone and 2-propanol) were generated by usingAvogadro, the geometry of the molecules was optimized under Molecular Mechanics force field (MMFF). Critical diameter is calculated by the diameter of the smallest cylinder that can be drawn around the molecule in its lowest energy conformer. The critical diameter of all molecules are shown in Figure S7.

Supplementary Text

Characterization

#1 Field emission scanning electron microscopy (FE-SEM)

The FE-SEM (Zeiss Ultraplus Thermal Field Emission Scanning Electron Microscope) image of 5A zeolite crystals were shown in Figure S1. 5A zeolite crystals are cubic and have an average particle size of approximately 2 µm. A proper concentration of 5A zeolite was dispersed in DI water and sonicated for 1 h and then dripped on the conductive carbon tape.

#2 X-ray photoelectron spectroscopy (XPS) analysis

The surface chemical compositions of 5A zeolite and MLD coated 5A zeolite were analyzed by XPS (Kratos Axis Ultra DLD instrument equipped with a monochromated Al Ka x-ray source and hemispherical analyzer capable of an energy resolution of 0.5 eV). As the MLD alucone coating thickness increased, much smaller amount underlying silicon can be seen (Figure S2).The Al and Si atomic concentration and Al/Si ratio was listed in Table 1.

#3 X-ray diffraction (XRD)

X-ray powder diffraction (XRD) was carried out using a Rigaku MiniFlex II diffractometer with Cu Kα radiation (λ = 0.15418 nm). The diffraction data was recorded for 2θ angles between

5° and 50°. XRD pattern was shown in Figure S3. XRD confirmed LTA zeolite structure before and after MLD, and suggested MLD coatings were amorphous.

#4 BET measurements

The BET surface areas were measured by a Micromeritcs ASAP 2020 unit. Prior to analysis, the materials were degassed in situ for 10 h at 250°C. The BET surface was calculated from the BET equation in the 0.05-0.3 relative pressure range and shown in Figure 1C.

#5 N_2 and Ar sorption analysis

N_2 adsorption isotherms at 77 K were measured on a Micromeritcs ASAP 2020 unit. Prior to analysis, the materials were degassed in situ for 10 h at 250°C. The micropore volume was analyzed by the t-plot. Pore size distribution was also calculated using adsorption branch of the isotherms. The results were shown in Figure S4. Figure S4a showed that the N_2 adsorption isotherms of 5A zeolite, 5A-Zeolite-60, and 5A-Zeolite-120 were almost the same. Figure S4b showed micropore volume for 5A zeolite, 5A-Zeolite-60, and 5A-Zeolite-120 are 0.195, 0.208, and 0.203 cm^3/g, respectively. This is consistent that the MLD coating is only deposited outside 5A zeolite surface. Figure S4c showed pore size distributions of 5A zeolite, 5A-Zeolite-60, and 5A-Zeolite-120, which were all centered at approximately 0.5 nm. This suggests MLD coating has similar pore size as 5A zeolite pores. Also, from pre-adsorption experiment (Fig. 3b), we concluded that MLD coating pore size is between 0.49 and 0.63 nm. This is consistent with the pore size distribution calculated from N_2 isotherms. In a very recent study from our group,[3] we deposited TiO_2 coating on 5A surface by MLD and calculated MLD coating pore size distribution using N_2 adsorption isotherms. We found a peak for TiO_2 MLD pores at ~1.1 nm, suggesting the sensitivity of using N_2 isotherms to calculate pore size distribution of MLD coating. This again supports our above conclusion that MLD coating pores are similar to 5A pores and there is no pores larger than 1 nm in the Al_2O_3 MLD coatings. Ar adsorption isotherms were also measured at 77 K (Figure S4d). The result again showed very similar Ar adsorption isotherms for 5A zeolite and 5A-Zeolite-60. The micropore volumes calculated by t-plot were 0.177 and 0.175 cm^3/g for 5A zeolite and 5A-Zeolite-60 (Figure S4e). This again shows that the micropore volume was not changed after MLD coating deposition. Micropore volume calculated by using Ar is 10~15% less than that of N_2 (0.195 and 0.208 cm^3/g). This trend is also reported by Raj[4] for the micropore volume calculation for 13X zeolite when using N_2 and Ar.

#6 Transmission electron microscopy (TEM)

The samples were dispersed in ethanol, dropped ono a carbon-coated copper grid, and dried. The TEM (Hitachi H8000 Scanning Transmission Electron Microscope) images of MLD coated 5A zeolite were shown in Figure 1a and Figure S5. Figure S5a and b showed the TEM images of 5A-Zeolite-60. A uniform ~20 nm thick MLD coating was deposited on the outside surface of 5A zeolite crystal. Figure S5c showed ~10 nm thick MLD coating on the 5A zeolite outside surface deposited by 30 cycles of MLD. This is consistent with the layer-by-layer growth mechanism of MLD process.

Table S1. Surface atomic concentrations of Al, Si, and O of 5A zeolite and 5A zeolite with different cycles of MLD coatings (after calcination), measured from XP spectra of Al 2P, Si 2P and O 1S.

**Cycles of MLD on 5A zeolite**	**Atomic concentration (%)**			**Al/Si ratio**
	**Al**	**Si**	**O**	
0	15.9	16.0	68.1	1.0
20	22.6	10.2	67.2	2.2
60	29.4	2.5	68.1	11.7
120	32.3	0.7	67.0	46.1
180	33.0	0.3	66.7	110

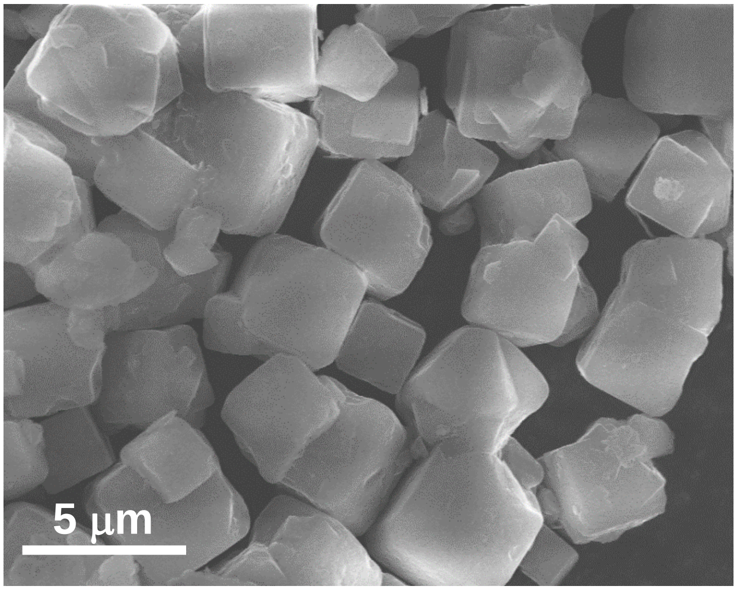

Figure S1: FE-SEM image of 5A zeolite.

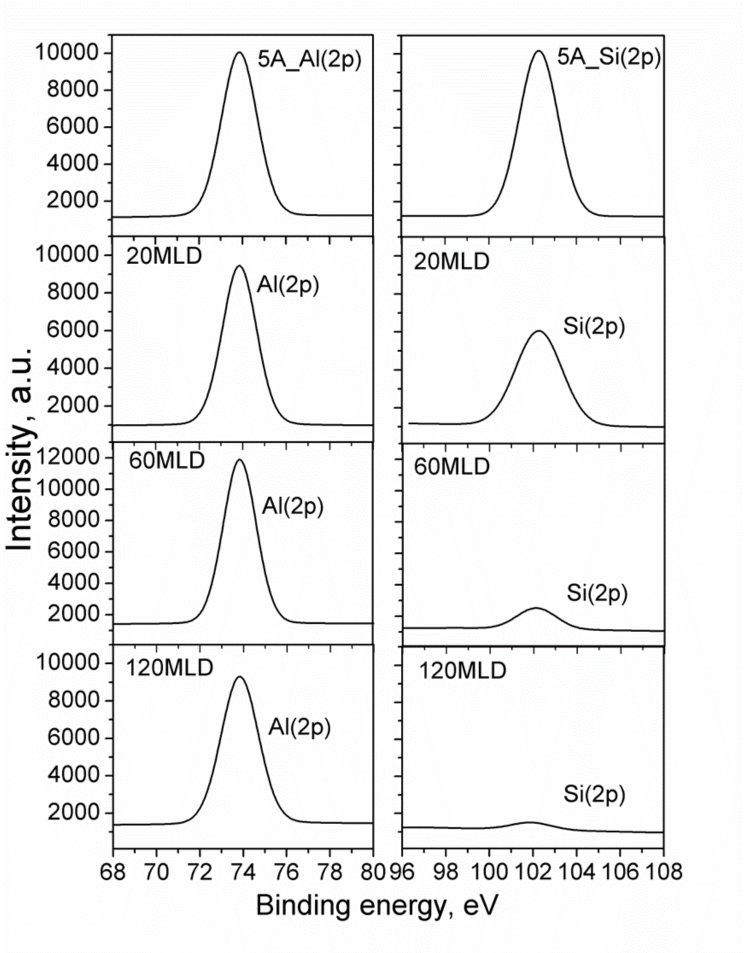

Figure S2: XP spectra of Al 2P and Si 2P on 5A zeolite and 5A zeolite with different cycles of MLD coatings (after calcination).

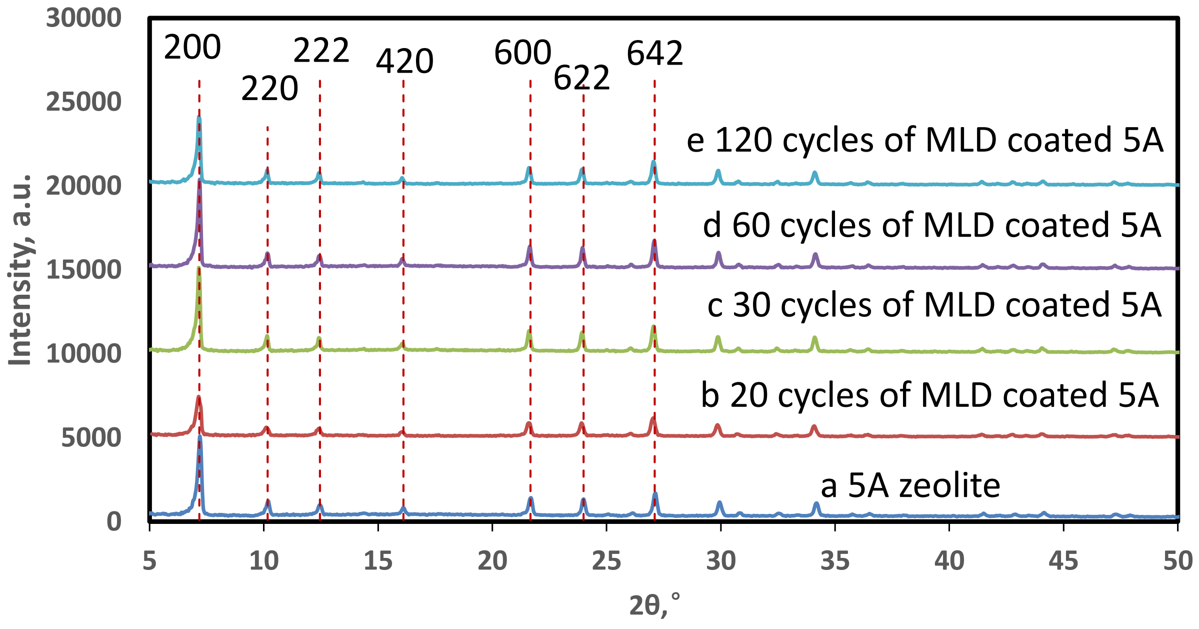

Figure S3: XRD patterns of 5A zeolite and 5A zeolite with different cycles of MLD coatings.

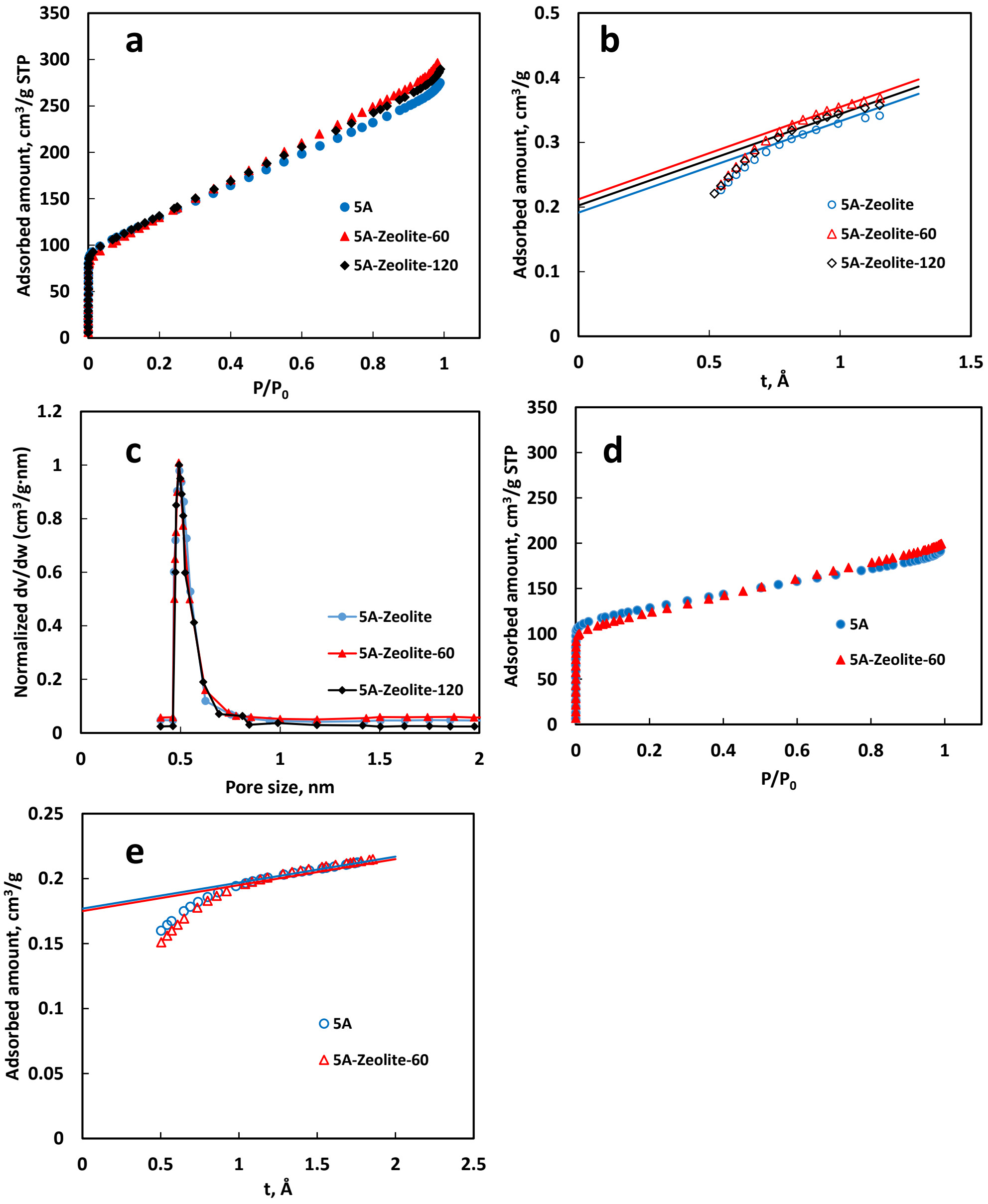

Figure S4: N_2 and Ar sorption analysis on 5A zeolite and 5A zeolite with different cycles of MLD coatings: (a) N_2 adsorption isotherms of 5A zeolite, 5A-Zeolite-60, and 5A-Zeolite-120 at 77 K; (b) t-plots for 5A zeolite, 5A-Zeolite-60, and 5A-Zeolite-120 calculated by using N_2; (c) Pore size distribution for 5A zeolite, 5A-Zeolite-60, and 5A-Zeolite-120; (d) Ar adsorption isotherms of 5A zeolite, and 5A-Zeolite-60 at 77 K; (e) t-plots for 5A zeolite and 5A-Zeolite-60 calculated by using Ar.

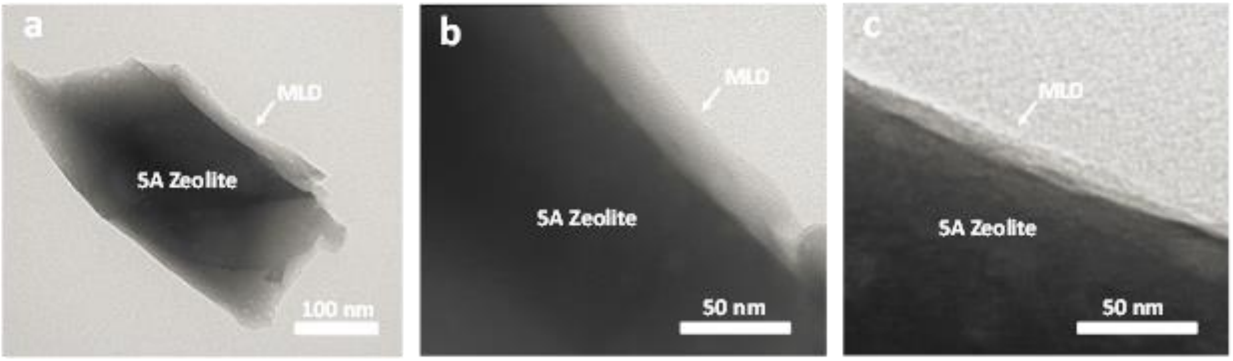

Figure S5: TEM images of 5A-Zeolite-60 at low (a) and high (b) magnification; (c) 5A-Zeolite-30.

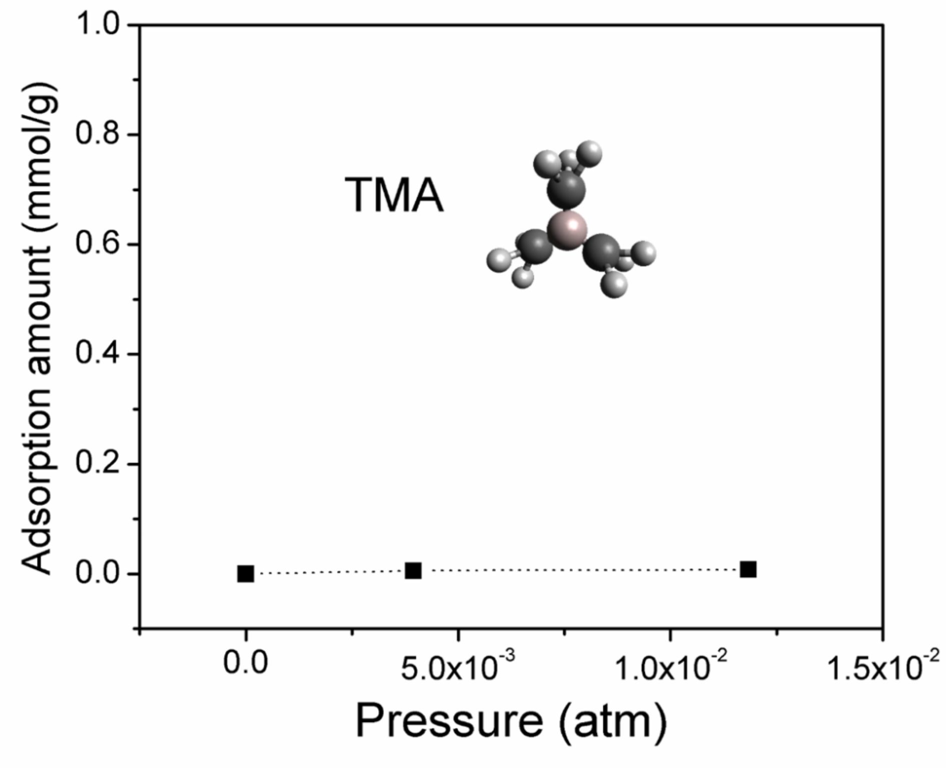

Figure S6: Vapor adsorption isotherm of TMA on 5A zeolite at 20℃.

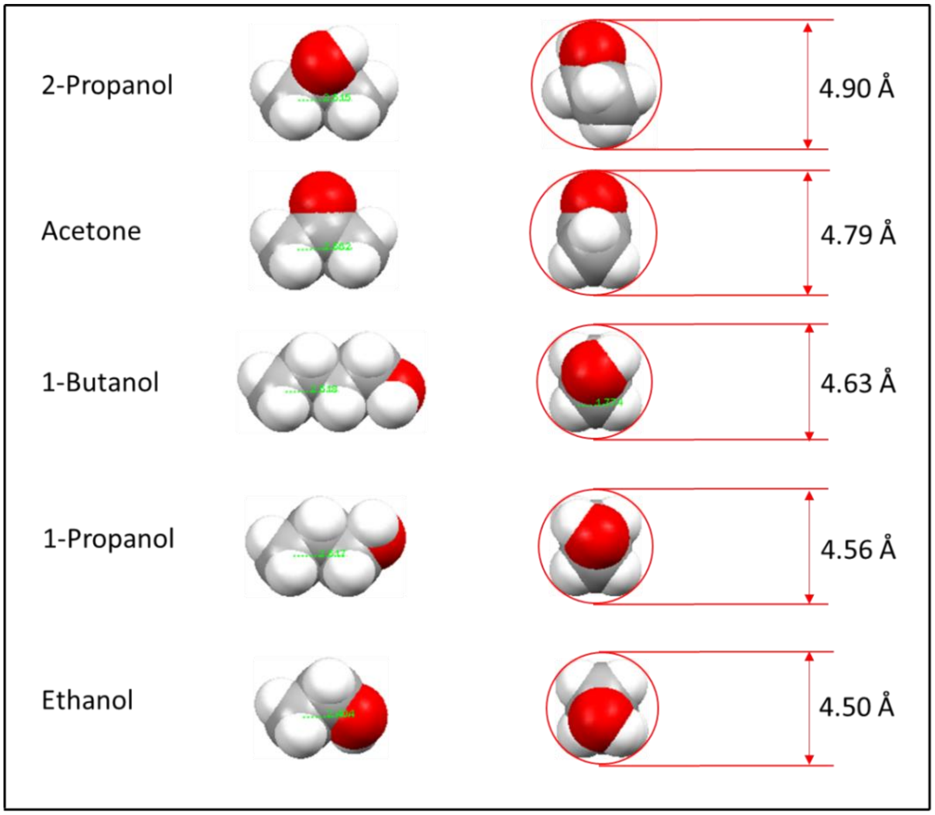

Figure S7: Molecular structures and critical diameters of 2-propanol, acetone, 1-butanol, 1-propanol and ethanol. Carbon atom: grey; oxygen atom: red; hydrogen atom: white.

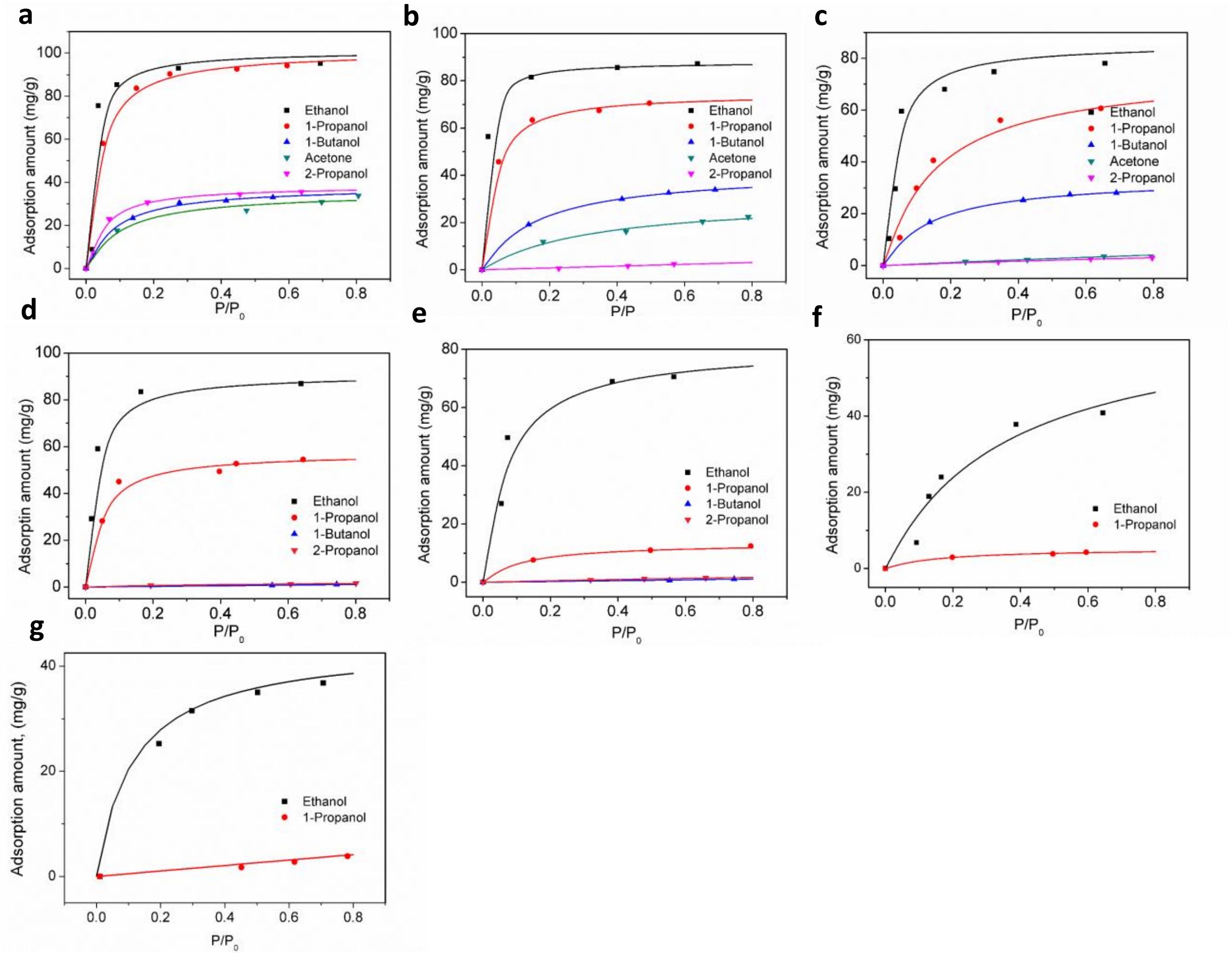

Figure S8: Adsorption isotherms of ethanol, 1-propanol, 1-butanol, acetone and 2-propanol at 20℃ on **(**a) 5A zeolite, (b) 5A-Zeolite-20, (c) 5A-Zeolite-30, (d) 5A-Zeolite-60, (e) 5A-Zeolite-120, (f) 5A-Zeolite-180, and (g) 5A-Zeolite-300. P is the vapor pressure, and P_0 is the saturation pressure. $P_{0(Ethanol)}$=50 Torr, $P_{0(1\text{-}Propanol)}$=18 Torr, $P_{0(1\text{-}Butanol)}$=7 Torr, $P_{0(Acetone)}$=201 Torr, $P_{0(2\text{-}Propanol)}$=36 Torr.

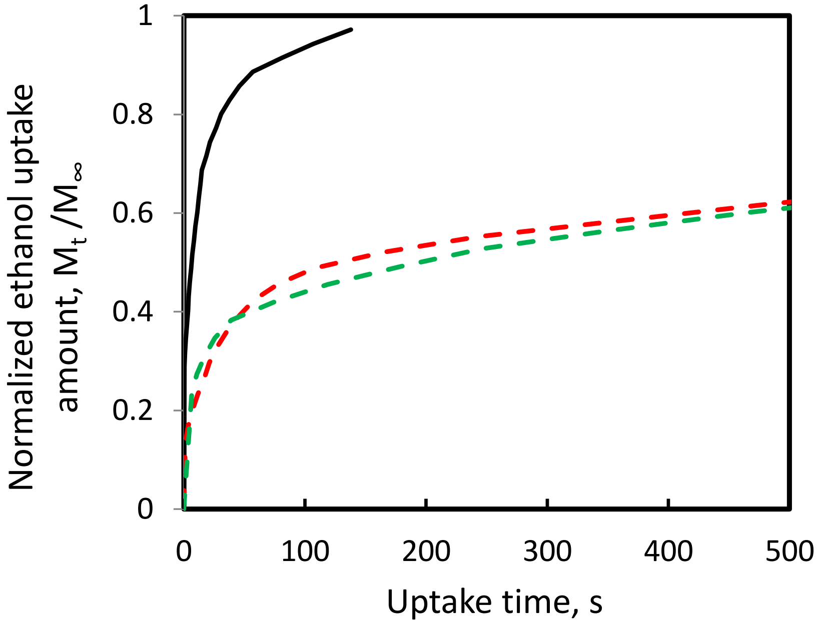

Figure S9: Ethanol adsorption kinetics on 5A zeolite (solid black line) and 5A-Zeolite-30 (dash red line) and 5A-Zeolite-60 (dash green line). M_t is adsorbed amount of ethanol at time t, and M_∞ is adsorbed amount at equilibrium.

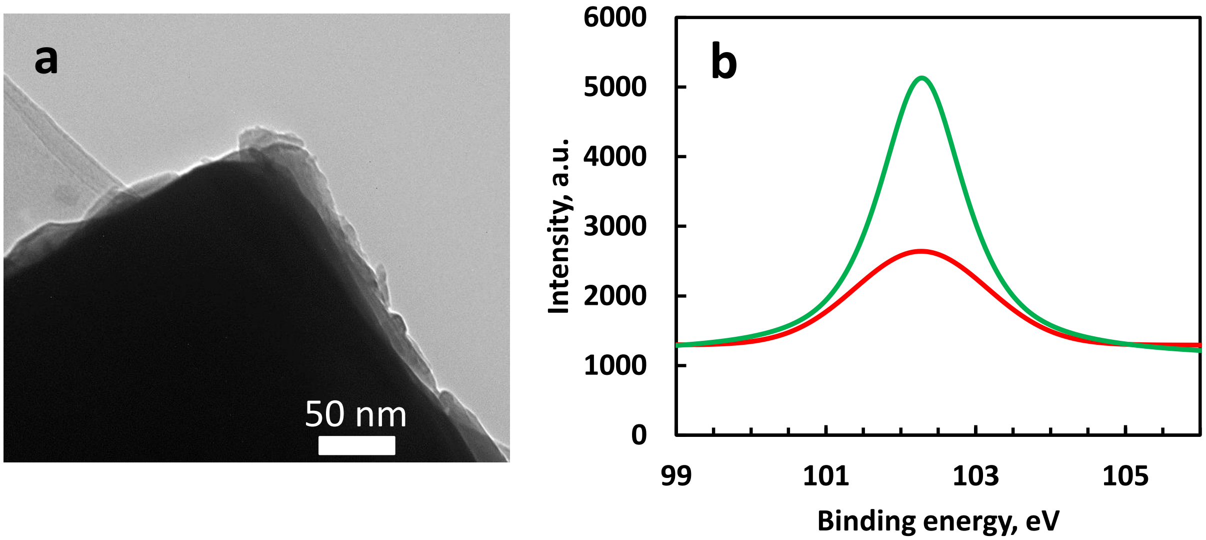

Figure S10:: (a) TEM image of 5A-Zeolite-60C; (b) XP spectra of Si 2P on 5A zeolite with 60 cycles MLD before (red) and after (green) crushing.

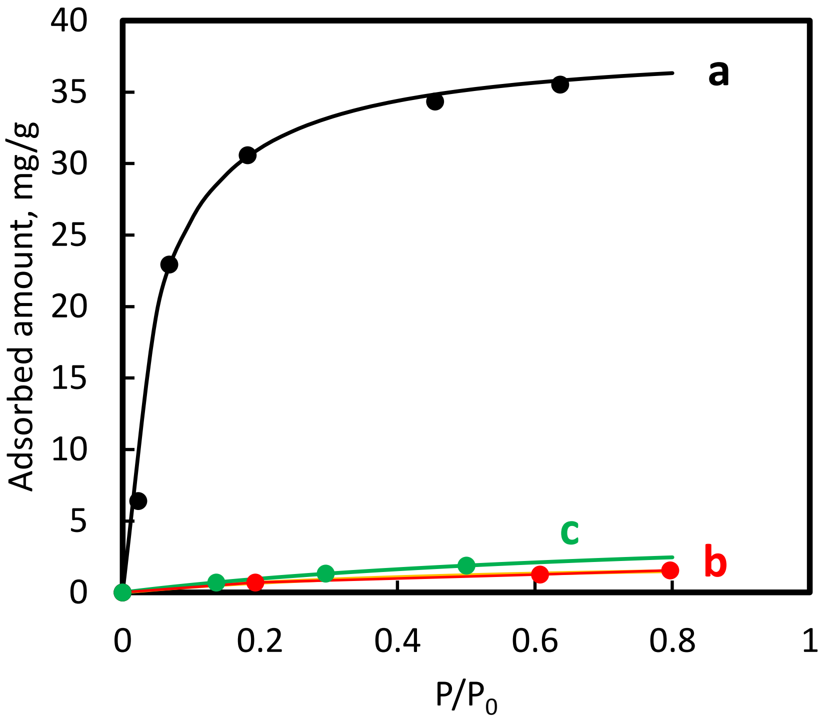

Figure S11: Adsorption isotherms of 2-propanol at 20℃ on **(**a) 5A-zeolite, (b) 5A-Zeolite-60, and (C) 5A-Zeolite-60C. P is the vapor pressure, and P_0 is the saturation pressure, $P_{0(2\text{-Propanol})}$=36 Torr.

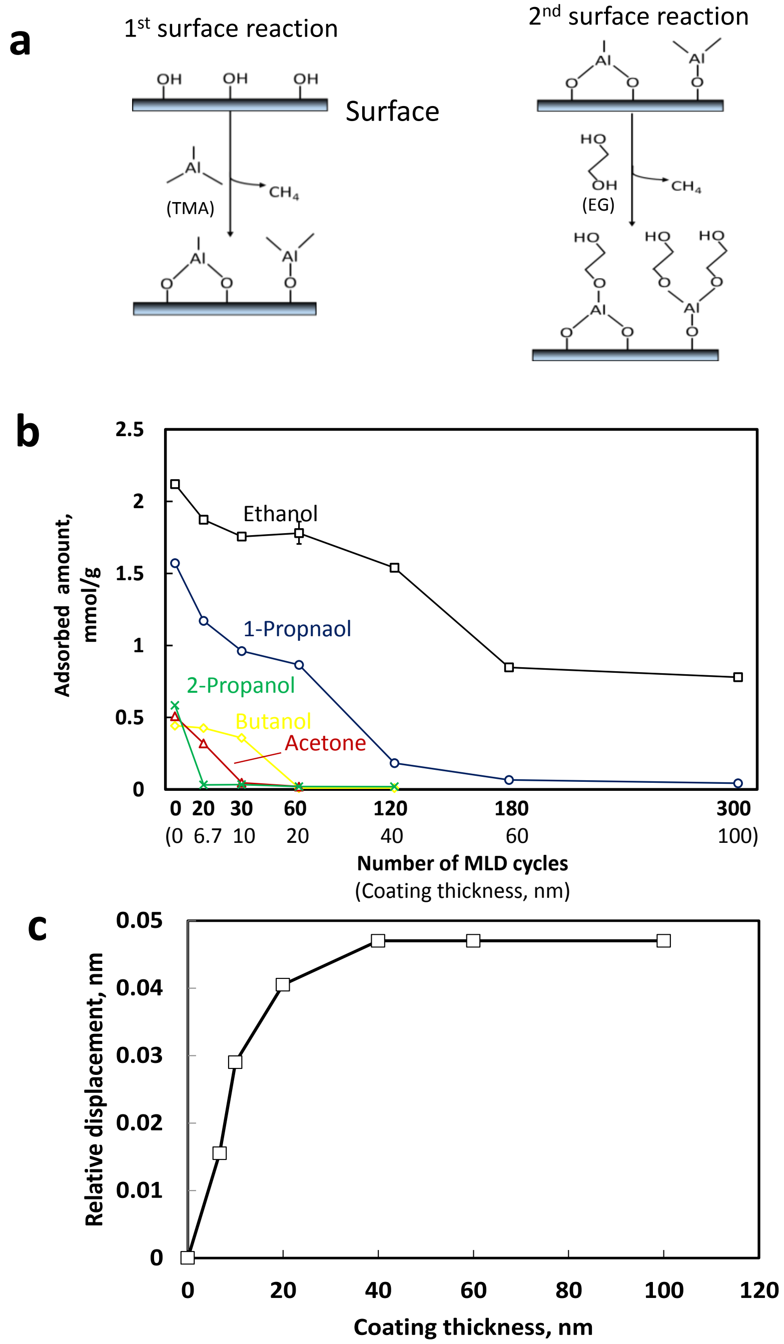

Figure S12: Analysis of MLD coatings on zeolite surface and pore misalignment with different MLD coating thicknesses**:** (a) Two self-limiting surface reactions of alucone MLD on a substrate surface; (b) Adsorbed amount of molecules with different sizes on 5A zeolite with different cycles of MLD/coating thickness up to 300 cycles/100 nm; (c) Relative displacement of the microporous Al_2O_3 coating on 5A zeolite surface vs. thickness of the coating; relative displacement is defined as the size difference between molecular "gate" and the 5A zeolite pore opening (~0.5 nm).

MLD on 5A zeolite surface is expected to form a uniform, hybrid alucone coating, whose morphology and composition are expected to be independent of the coating thickness due to the self-limiting feature of the two surface reactions (Figure S12a) and the same substrate surface/hydroxyl group distribution and density. In addition, bonding of the alucone with 5A zeolite at the interface is also expected to be the same. During calcination, carbon compound will be removed, and the micropores will be generated in the MLD coating and the whole coating will shrink approximately 1/3, as reported before[5]. At the same time, interfacial shear force between the microporous Al_2O_3 coating and 5A zeolite will be generated due to the different thermal expansion coefficients of alumina[6] and zeolite[7]. According to an analytical model based on continuum mixture theories, Nassar *et al*.[8] found that the interfacial thermal stress between two contacting materials increases with the increase of the adhesive/coating thickness; as a result, thicker coatings may lead to larger shift/relative displacement between two contacting materials. Figure S12b shows that the molecular "gate" size decreases with the increase of the coating thickness when coating is thinner than 40 nm, suggesting a continuous shift with the increase of the coating thickness. This is consistent with the modelling results. However, when the resistance, namely chemical bonding between the MLD coating and 5A zeolite surface, balances the thermal shear force, the relative movement between the Al_2O_3 coating and 5A zeolite surface stops and thus no obvious decrease of the molecular "gate" size was detected for coatings thicker than 40 nm. Figure S12c summarizes the relative shift/displacement between the microporous MLD coating and 5A zeolite surface, which clearly reflects the above discussed trend.

References

1 Gumma, S. & Talu, O. Gibbs dividing surface and helium adsorption. *Adsorption* **9**, 17-28 (2003).

2 Karger, J. & Pfeifer, H. NMR self-diffusion studies in zeolite science and technology. *Zeolites* **7**, 90-107 (1987).

3 Song, Z. N., Huang, Y., Wang, L., Li, S. G. & Yu, M. Composite 5A zeolite with ultrathin porous TiO2 coating for selective gas adsorption. *Chem. Commun.* **51**, 373-375 (2015).

4 Vyas, R. K., Shashi & Kumar, S. Determination of micropore volume and surface area of zeolite molecular sieves by D-R and D-A equations: A comparative study. *Indian J. Chem. Technol.* **11**, 704-709 (2004).

5 Liang, X. H., Yu, M., Li, J. H., Jiang, Y. B. & Weimer, A. W. Ultra-thin microporous-mesoporous metal oxide films prepared by molecular layer deposition (MLD). *Chem. Commun.*, 7140-7142 (2009).

6 Becher, P. F., Hsueh, C. H. & Waters, S. B. Thermal-expansion anisotropy in hot-pressed sic-whisker-reinforced alumina composites. *Mater. Sci. Eng., A* **196**, 249-251 (1995).

7 Noack, M., Schneider, M., Dittmar, A., Georgi, G. & Caro, J. The change of the unit cell dimension of different zeolite types by heating and its influence on supported membrane layers. *Microporous Mesoporous Mater.* **117**, 10-21 (2009).

8 Nassar, S. A. & Virupaksha, V. L. Effect of adhesive thickness and properties on the biaxial Interfacial shear stresses in bonded joints using a continuum mixture model. *J. Eng. Mater. Technol.* **131**, 9 (2009).
